# Supplementary material for: Synthesis of ZnO Nanoparticles Doped with Cobalt Using Bimetallic ZIFs as Sacrificial Agents
Source: Nanomaterials (Basel). 2020 Jun 30;10(7):1275. doi: 10.3390/nano10071275 (PMC7408057; doi:10.3390/nano10071275)
Supplement: Supplementary file 1 [file nanomaterials-10-01275-s001.pdf]

nanomaterials

Supplementary Material

Synthesis of ZnO nanoparticles doped with cobalt using bimetallic ZIFs as sacrificial agents

Vera V. Butova [1,*], Vladimir A. Polyakov [1], Elena A. Erofeeva [1], Sofia A. Efimova [1], Mikhail A. Soldatov [1], Alexander L. Trigub [2], Yury V. Rusalev [1] and Alexander V. Soldatov [1]

[1] The Smart Materials Research Institute, Southern Federal University, Sladkova str. 178/24, 344090 Rostov-on-Don, Russia; vlpolyakov@sfedu.ru (V.A.P.); bulanova@sfedu.ru (E.A.E.); sefimova@sfedu.ru (S.A.E.); mikhail.soldatov@gmail.com (M.A.S.); rusalev@sfedu.ru (Y.V.R.); soldatov@sfedu.ru (A.V.S.)

[2] National Research Centre, Kurchatov Institute, 1 Akademika Kurchatova pl, 123182 Moscow, Russia; alexander.trigub@gmail.com

* Correspondence: vbutova@sfedu.ru

1. Structure of ZIF-8

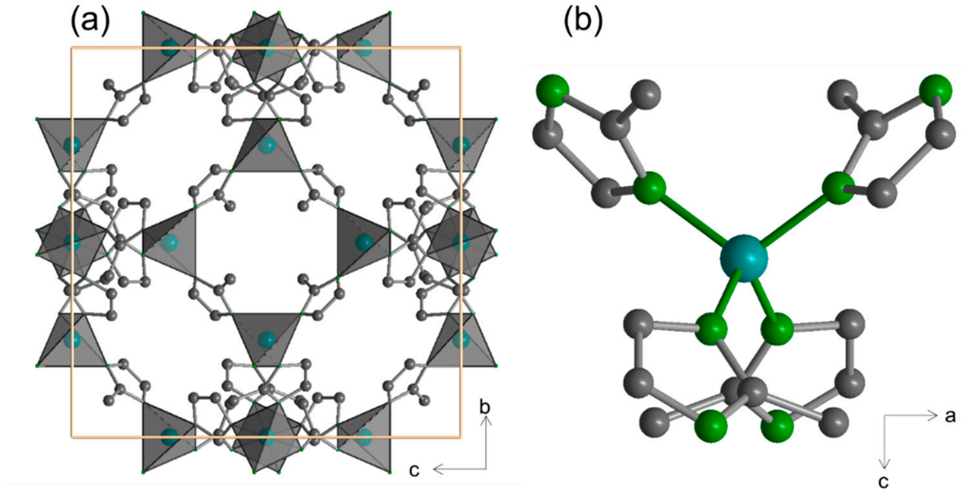

Figure S1. Polyhedral model of ZIF-8 structure (**a**) and scheme of coordination of zinc ions with linker molecules (**b**). Gray tetrahedra represent coordination of zinc with nitrogen, gray spheres stay for carbon, green ones denote nitrogen, blue ones show zinc. In right bottom corner crystallographic axes are provided.

2. Synthesis of Zn/Co-ZIF-8 precursors

Zinc and cobalt nitrates hexahydrates and 2-methyl imidazole (MIm) were dissolved separately in the equal volumes of dimethylformamide (DMF). Then triethylamine (TEA) was added to the linker, and both solutions were transferred into the glass vessel, closed hermetically, and placed into the microwave (MW) oven. Then, the reaction mixture was heated at 140 °C for 15 min with magnetic stirring. After cooling down to room temperature, a precipitate was collected by centrifugation, washed two times with DMF, and once with methanol and dried at 60 °C overnight.

Table S1. Amounts of precursors used for Zn/Co-ZIF-8 synthesis.

Sample designation	Molar ratio		$Zn(NO_3)_2·6H_2O$		$Co(NO_3)_2·6H_2O$		MIm		TEA		DMF	
	Zn^{2+}	Co^{2+}	*n*, (mmol)	*m*, (g)	*n*, (mmol)	*m*, (g)	*n*, (mmol)	*m*, (g)	*n*, (mmol)	*V*, (μL)	*n*, (mol)	*V*, (mL)
100Zn0Co-ZIF	100	0	0.4469	0.1329	0	0	1.7876	0.1466	1.1620	161.5	0.1292	10
99Zn1Co-ZIF	99	1	0.4424	0.1316	0.0045	0.0013	1.7876	0.1466	1.1620	161.5	0.1292	10
95Zn5Co-ZIF	95	5	0.4246	0.1263	0.0223	0.0065	1.7876	0.1466	1.1620	161.5	0.1292	10
75Zn25Co-ZIF	75	25	0.3352	0.0997	0.1117	0.0325	1.7876	0.1466	1.1620	161.5	0.1292	10
50Zn50Co-ZIF	50	50	0.2235	0.0665	0.2235	0.0650	1.7876	0.1466	1.1620	161.5	0.1292	10
0Zn100Co-ZIF	0	100	0	0	0.4469	0.1301	1.7876	0.1466	1.1620	161.5	0.1292	10

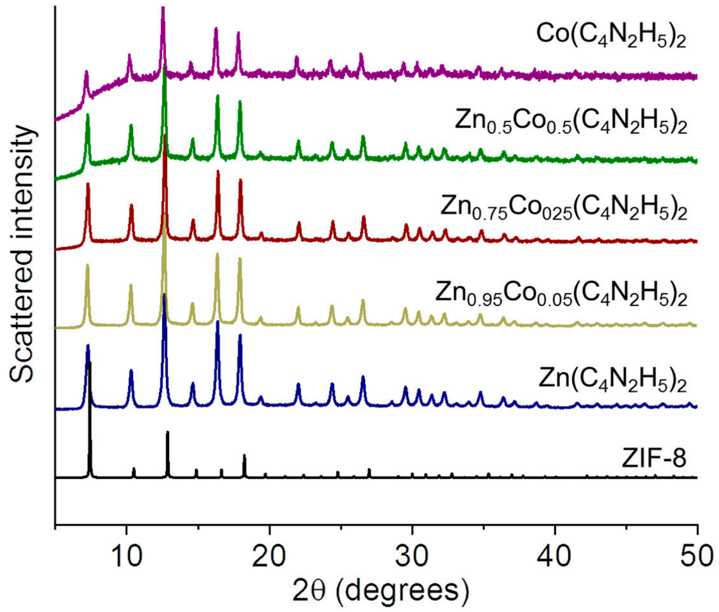

Figure S2. XRD patterns of ZIF-precursors. Pattern designated as ZIF-8 was calculated according to crystallographic data (COD 602542).

3. Crystal structure of ZnO and Co_3O_4

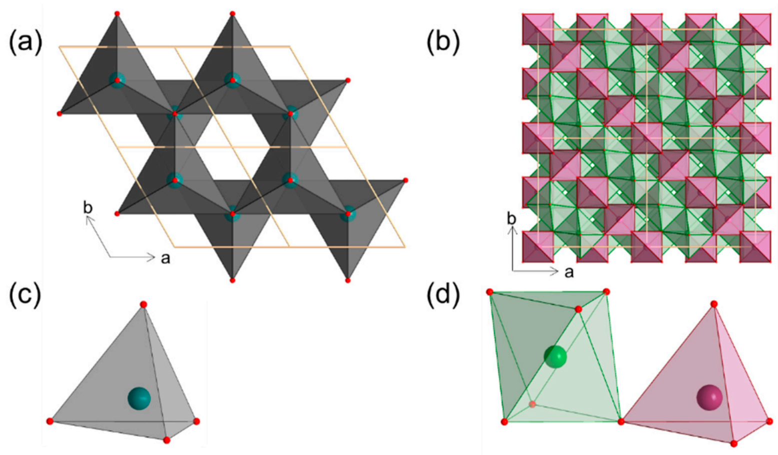

Figure S3. Polyhedral models of ZnO with wurtzite structure (**a**) and Co_3O_4 with spinel structure (**b**). Gray tetrahedra represent coordination of Zn^{2+} ions (**a**,**c**), pink tetrahedra show coordination of Co^{2+} ions, while green octahedra show coordination of Co^{3+} ions (**b**,**d**). Blue spheres represent Zn^{2+} ions, green – Co^{3+}, pink – Co^{2+}. In the left bottom corners of parts (**a**) and (**b**), crystallographic axes are provided.

4. X-ray diffraction

Part of the plots showed a slightly negative strain. We suppose that it could be assigned to the lattice shrinkage that was observed in the calculation of lattice parameters.

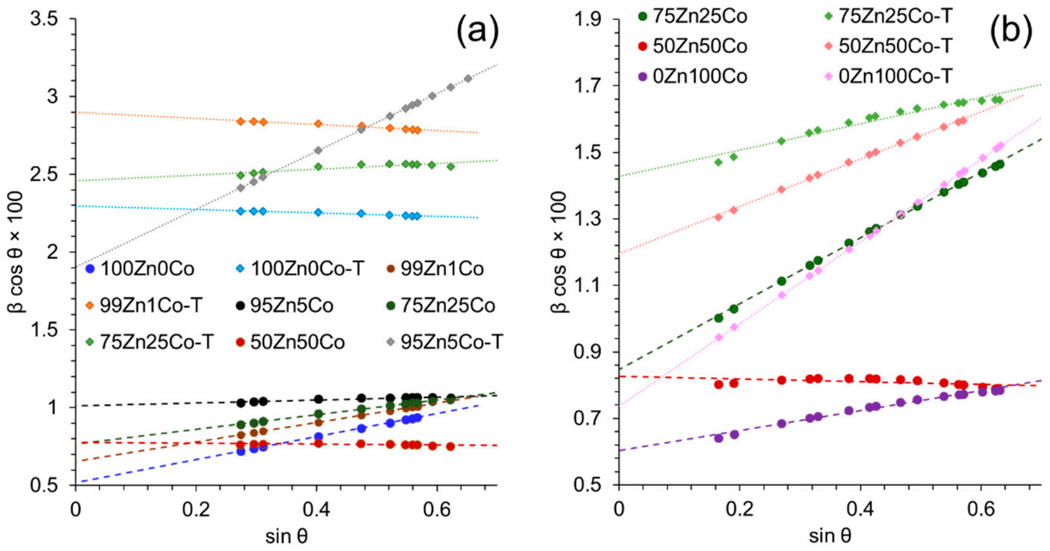

Figure S4. Plots calculated according to the Williamson-Hall method using data from XRD profile analysis in Jana2006. Part (**a**) represents data for hexagonal wurtzite phases, while part (**b**) corresponds to cubic spinel phases.

5. Transmission electron microscopy (TEM)

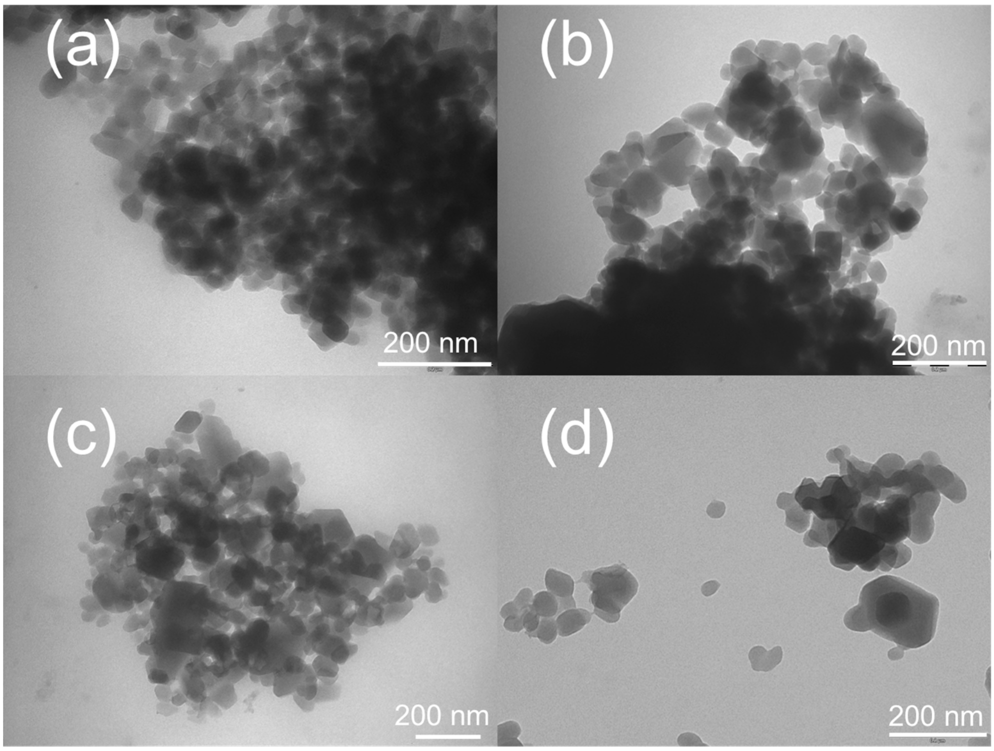

Figure S5. TEM images of ZIFs used as precursors for annealing: 100Zn0Co-ZIF (**a**), 50Zn50Co-ZIF (**b**), 75Zn25Co-ZIF (**c**), 0Zn100Co-ZIF (**d**).

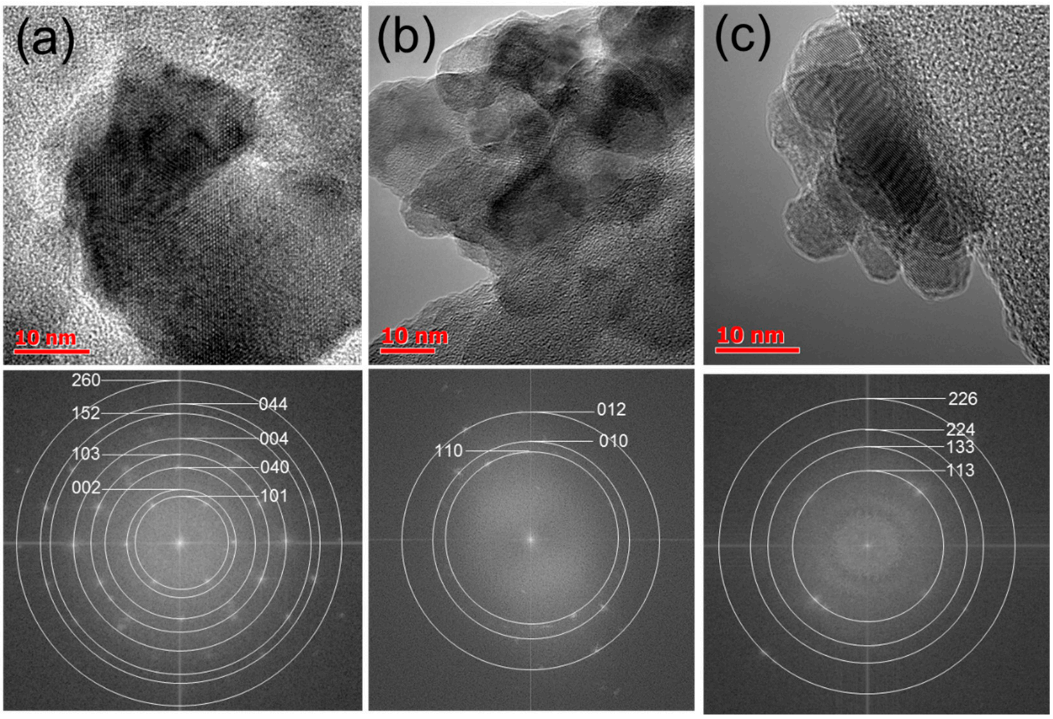

Figure S6. TEM images (above) and FFT (below) of the image with the contribution from selected reflections of samples 100Zn0Co-T (**a**), 95Zn5Co-T (**b**),0Zn100Co-T (**c**). Reflections of the 100Zn0Co-T sample correspond to Zn2SiO4 (COD 1549039).

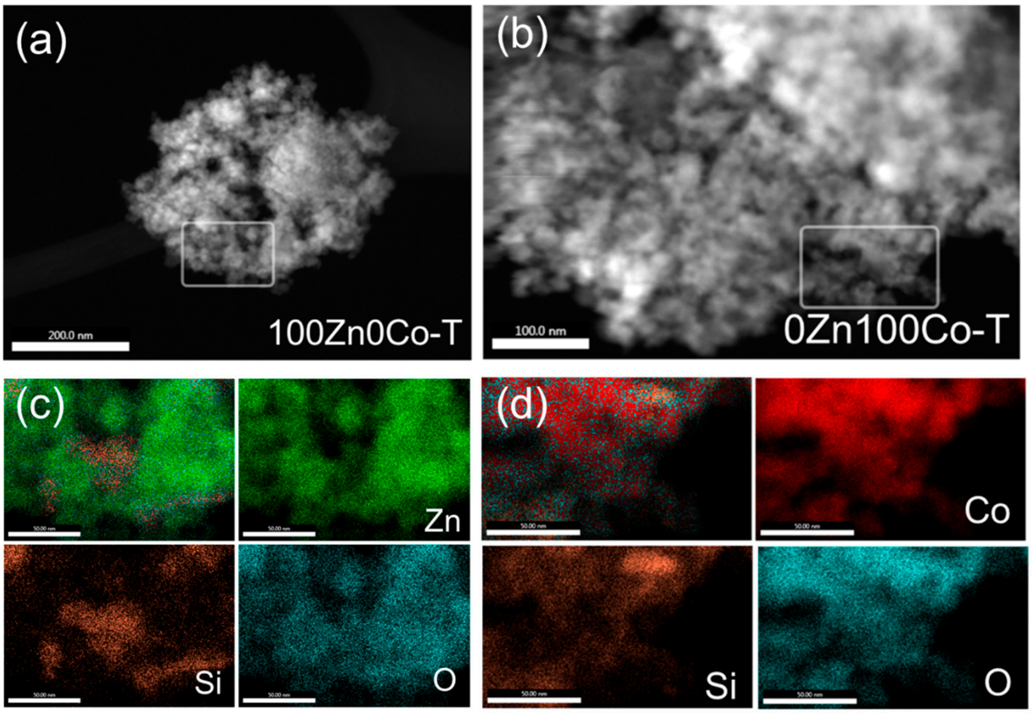

Figure 7. TEM images of samples 100Zn0Co-T (**a**) and 0Zn100Co-T (**b**). EDX mapping for samples 100Zn0Co-T (**c**) and 0Zn100Co-T (**d**).

6. Magnetic Properties

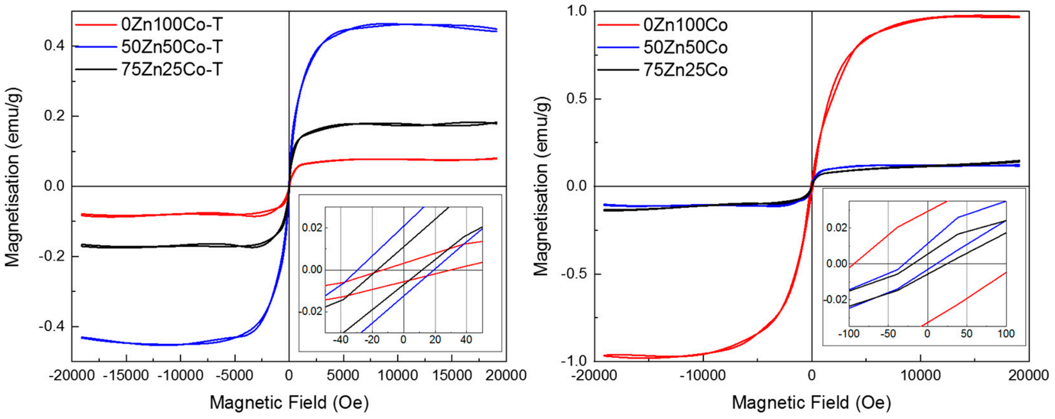

Figure S8. Magnetic moment vs. magnetic field for 0Zn100Co, 50Zn50Co, 75Zn25Co, 0Zn100Co-T, 50Zn50Co-T, and 75Zn25Co-T samples. Insets represent low magnetic field rang.

5. Nitrogen Adsorption

Table S2. Specific surface areas of samples 100Zn0Co, 50Zn50Co, 0Zn100Co, 100Zn0Co-T, 50Zn50Co-T, and 0Zn100Co-T. BET stands for Brunauer–Emmett–Teller.

	BET (m^2/g)	t-plot micropore area (m^2/g)	t-plot external area (m^2/g)
100Zn0Co-ZIF	1608	1505	103
95Zn5Co-ZIF	1359	1339	20
75Zn25Co-ZIF	1612	1574	38
50Zn50Co-ZIF	1642	1586	56
0Zn100Co-ZIF	1657	1605	52
100Zn0Co	12	1.4	10.9
100Zn0Co-T	45	8.8	36.1
50Zn50Co	18	3.8	14.1
50Zn50Co-T	46	14.6	30.9
0Zn100Co	11	-	11.3
0Zn100Co-T	101	11	89

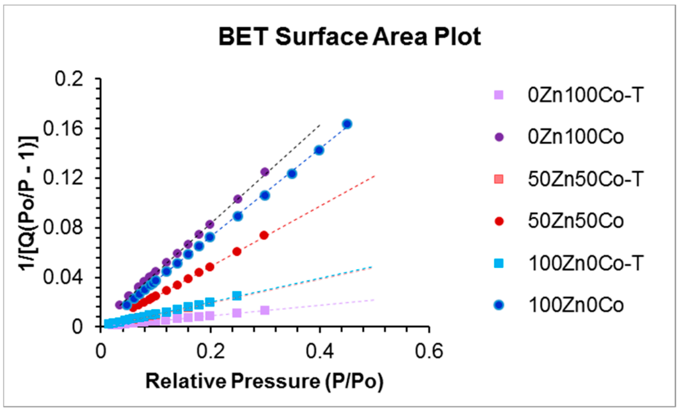

Figure S9. BET surface area plots for samples 100Zn0Co, 50Zn50Co, and 0Zn100Co (circle markers) and 100Zn0Co-T, 50Zn50Co-T, and 0Zn100Co-T (square markers).

© 2020 by the authors. Licensee MDPI, Basel, Switzerland. This article is an open access article distributed under the terms and conditions of the Creative Commons Attribution (CC BY) license (http://creativecommons.org/licenses/by/4.0/).
